# Investigation of the optimal method of oxygen administration with simultaneous use of a surgical mask in postoperative patients: a randomized cross-over study

**DOI:** 10.1186/s40981-024-00741-0

**Published:** 2024-09-28

**Authors:** Aya Kamiyama, Tomonori Takazawa, Yusuke Matsui, Kazuhiro Nagumo, Seiji Arai, Shigeru Saito

**Affiliations:** 1 Intensive Care Unit, Gunma University Hospital, 3-39-15 Showa-Machi, Maebashi, Gunma 371-8511 Japan; 2 Department of Anesthesiology, Faculty of Medicine, University of Toyama, 2630 Sugitani, Toyama, 930-0194 Japan; 3 Department of Anesthesiology, National Hospital Organization Takasaki General Medical Center, 36 Takamatsu-Cho, Takasaki, 370-0829 Japan; 4 Department of Anesthesiology, Gunma University Graduate School of Medicine, 3-39-22 Showa-Machi, Maebashi, Gunma 371-8511 Japan; 5 Department of Urology, Gunma University Graduate School of Medicine, 3-39-22 Showa-Machi, Maebashi, Gunma 371-8511 Japan

**Keywords:** Oxygen administration, Surgical mask, Oxygen mask, Nasal cannula, COVID-19

## Abstract

**Background:**

From the standpoint of infection prevention, anesthesiologists need to simultaneously use a surgical mask and an oxygen mask when administering oxygen to patients. However, there is a lack of scientific evidence to justify this method. We aimed to investigate a suitable method of oxygen administration when using a surgical mask in postoperative patients.

**Methods:**

This was a randomized, single-blind, cross-over study involving 42 patients admitted to the ICU. We compared three methods of oxygen administration: nasal cannula under the surgical mask, oxygen mask under the surgical mask, and oxygen mask above the surgical mask, using a cross-over design. The primary endpoint was partial pressure of arterial oxygen (PaO_2_). The secondary endpoint was partial pressure of arterial carbon dioxide (PaCO_2_).

**Results:**

PaO_2_ was higher when the oxygen mask was placed under the surgical mask (median values 197.7 mmHg), the nasal cannula was under the surgical mask (180.6 mmHg), and the oxygen mask was above the surgical mask (143.0 mmHg), in descending order, with significant differences between all groups (*P* < 0.001). PaCO_2_ did not differ between groups.

**Conclusions:**

The current standard method of administering oxygen to postoperative patients using an oxygen mask over a surgical mask results in poor oxygenation. Adopting the method of oxygen administration under the surgical mask via an oxygen mask or nasal cannula should be considered instead.

**Supplementary Information:**

The online version contains supplementary material available at 10.1186/s40981-024-00741-0.

## Introduction

The use of surgical masks for patients with suspected or proven respiratory infections is recommended to prevent nosocomial infections [1, 2]. Indeed, surgical masks have been reported effective in preventing expelled air dispersion [3] and viral infections [4]. Recently, surgical masks have been widely used in COVID-19 patients requiring oxygen administration. In relation to anti-COVID-19 measures, multiple organizations have recommended that oxygen be administered over the surgical mask when administering oxygen to a patient after general anesthesia [5, 6]. The use of surgical masks in medical settings is expected to continue even now, after the end of the COVID-19 pandemic.

Although the use of surgical masks in post-general anesthesia patients is widespread, their effect on oxygenation is controversial. Various reports have shown contradictory results, stating both that oxygen administered over a surgical mask does not affect oxygenation [7], and that it decreases oxygenation [8–12]. We previously compared three methods of oxygen administration utilizing a surgical mask in a study of healthy volunteers: (1) oxygen administered with an oxygen mask over a surgical mask, (2) oxygen administered with an oxygen mask under a surgical mask, and (3) oxygen administered via nasal cannula under the surgical mask. Among these methods, administering oxygen via a nasal cannula placed under the surgical mask provided the best oxygenation, followed by oxygen administration with an oxygen mask placed under the surgical mask and oxygen administration with an oxygen mask placed above the surgical mask [10]. Our previous study suggested that oxygenation might be better with a nasal cannula than with an oxygen mask. However, it was unclear whether the same results would be achieved in postoperative patients. In addition, since our previous study used the oxygen reserve index (ORi) to estimate the partial pressure of arterial oxygen (PaO_2_), more accurate assessments of oxygenation by measuring PaO_2_ levels were needed.

In the present study, we compared oxygenation with three oxygen administration methods using a surgical mask in urological patients who were admitted to the ICU after elective surgery. Oxygenation was assessed using PaO_2_ measurements.

## Methods

### Study design and ethics approval

This randomized, single-blind, cross-over study was performed at the intensive care unit of Gunma University Hospital from December 15, 2021, to July 26, 2022. This study conformed to the standards of the Declaration of Helsinki and was approved by the ethics committee of Gunma University Hospital (Trial No. IRB2021-042) on November 25, 2021. The study was registered with the University Hospital Medical Information Network Clinical Trials Registry (UMIN000046280) on December 6, 2021. Study participation was voluntary, and each patient gave their written informed consent for participation.

### Subjects

Inclusion criteria for study participation were (1) post-urologic surgery patients over 20 years of age scheduled for elective admission to the ICU after surgery, (2) legally competent to consent, and (3) scheduled for intraoperative and postoperative management with direct measurement of arterial pressure via an arterial cannula. Exclusion criteria were (1) history of respiratory illness, (2) American Society of Anesthesiologists physical status three or higher, and (3) determined by the physician as being ineligible for participation.

The criteria for discontinuation of the research were: (1) need for discontinuation, as judged by the physician in charge, due to the development of undesirable symptoms, (2) withdrawal of consent by the research subject, (3) decrease in the patient’s percutaneous oxygen saturation (SpO_2_) to below 91%, (4) decrease in PaO_2_ to below 60 mmHg, (5) increase of partial pressure of arterial carbon dioxide (PaCO_2_) to 55 mmHg or higher, (6) patient remaining intubated at the time of admission to the ICU, (7) cancellation of ICU admission, and (8) if the patient met any of the exclusion criteria.

Based on our pilot study, the required sample size was 32 subjects with power analysis using α = 0.05 and β = 0.8. Assuming a 25% dropout rate, 42 participants were enrolled in the present study.

### Endpoints

The primary endpoint was PaO_2_ level five minutes after the change in oxygen delivery method, and the secondary endpoints were PaCO_2_ levels at the same time points.

### Oxygen administration method

The intervention was initiated when more than one hour had elapsed since the patient’s admission to the ICU and when the administration of 4 L.min^−1^ of oxygen was considered sufficient. Oxygen was administered at a flow rate of 4 L.min^−1^ with an oxygen mask (Japan Medicalnext Co. Ltd., Osaka, Japan, catalog No. 1135015) or oxygen cannula (Japan Medicalnext Co. Ltd., Osaka, Japan, catalog No. 001597). COMFORT + ® Level-1 (Medicom Japan Inc. Ltd., Kobe, Japan), which is the surgical mask we use in our daily practice and meets level 1 of the American Society for Testing and Materials (ASTM), was used as the surgical mask. This mask has a particle filtration efficacy (PFE), which is an indicator of the ability to collect particles 0.1 μm in diameter, of higher than 98%, indicating that it has sufficient performance to collect small particles.

**Fig. 1:**
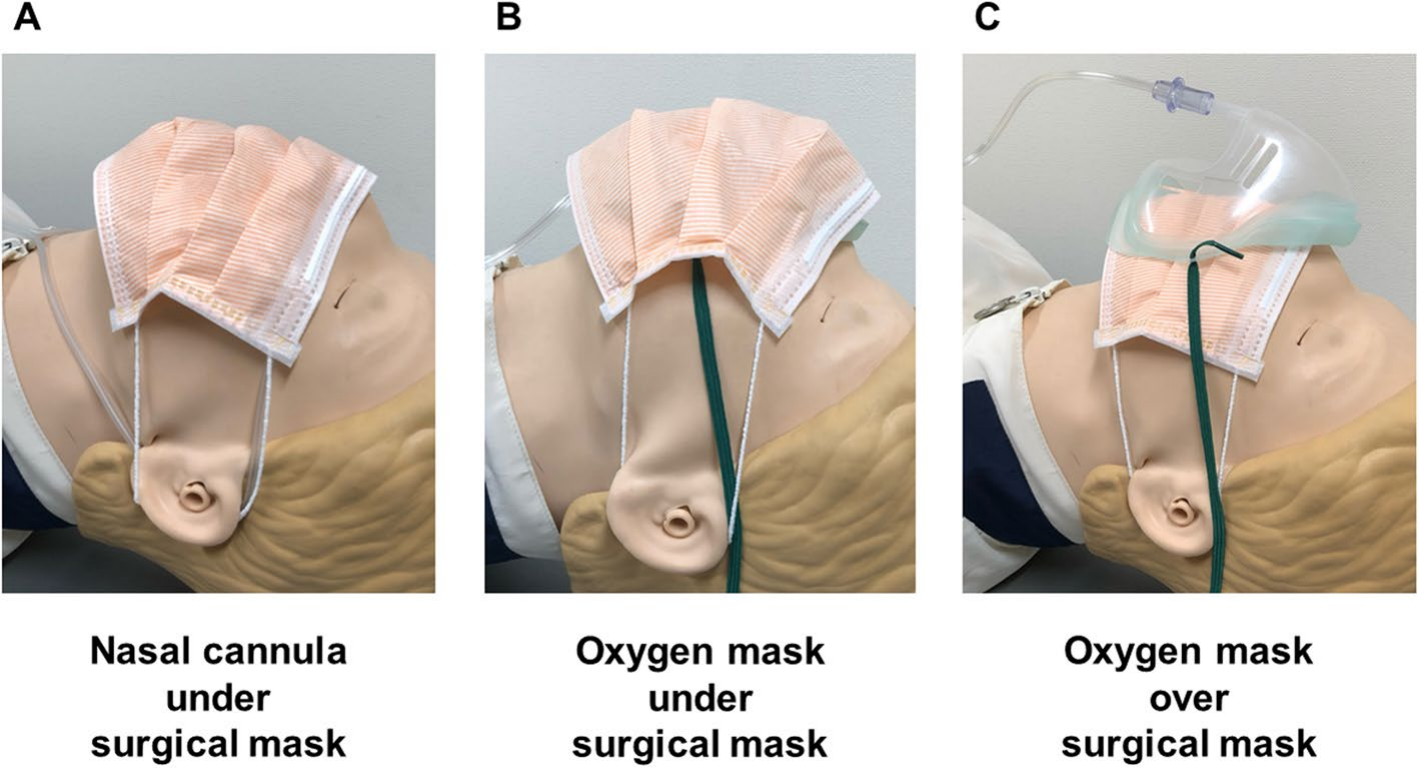
The three different oxygen administration methods tested. The images show wearing a nasal cannula under the surgical mask (**A**), and wearing an oxygen mask under (**B**) and over (**C**) the surgical mask

**Fig. 2:**
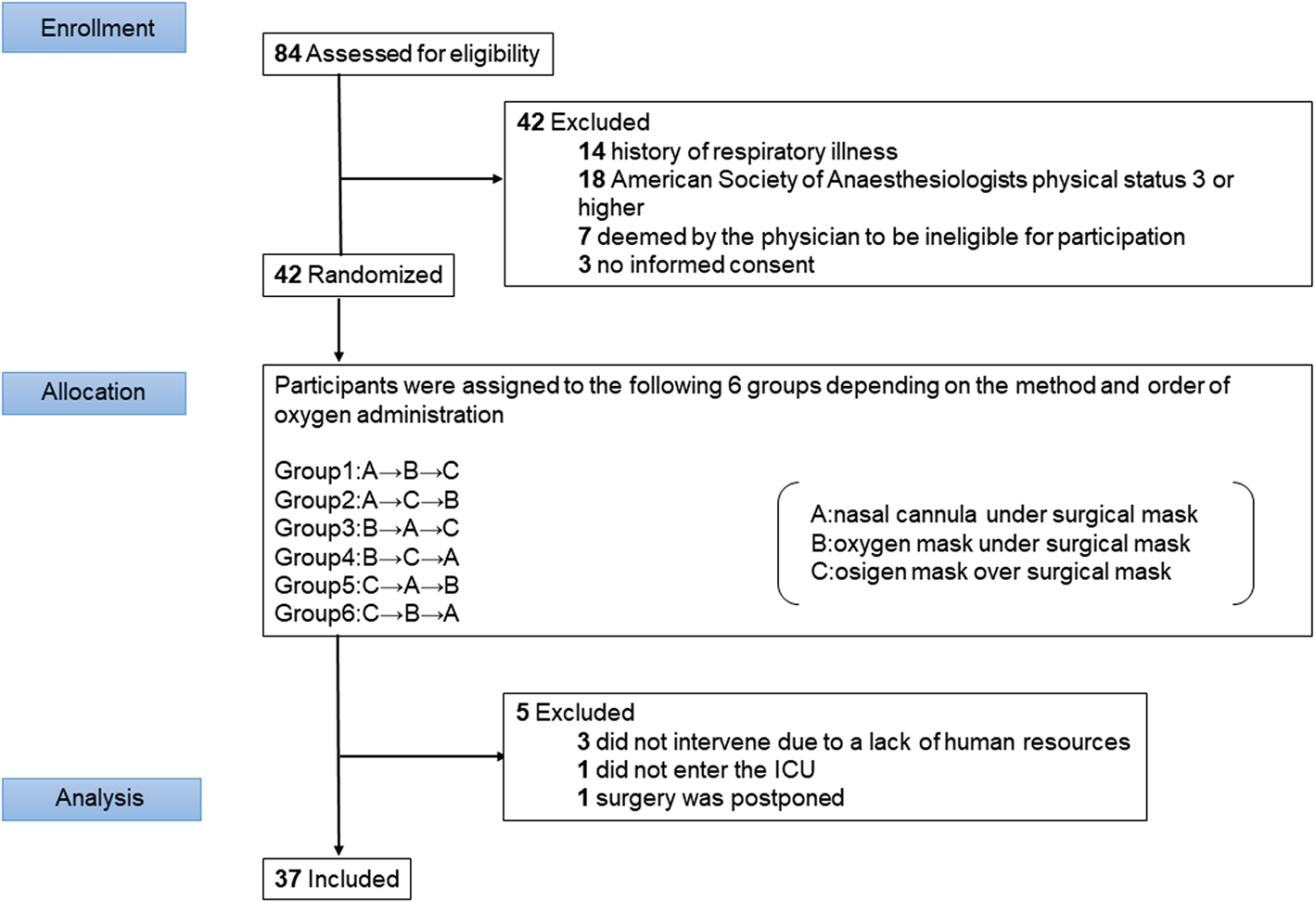
Subject recruitment, randomization, and analysis

Using the cross-over method, oxygen was sequentially administered by each of the following three methods (Fig. 1): via nasal cannula under the surgical mask, with an oxygen mask under the surgical mask, and via an oxygen mask over the surgical mask. All participants received oxygen once by each method. The order of administration was randomly selected from among six different ways using the envelope method (Fig. 2). In the envelope method, a piece of paper with the order of oxygen administration was placed inside the envelope, and oxygen was administered in the order indicated on the paper. This randomization allowed us to eliminate the potential risk of systematic error that might arise from fixing the order of oxygen administration methods. Randomization and patient assignment were performed by a single investigator (A.K.).

### Measurement method

Five minutes after changing the oxygen administration method, arterial blood was collected via the arterial catheter, and PaO_2_ and PaCO_2_ were measured using a blood gas analyzer (ABL800FLEX® Radiometer Corporation, Tokyo, Japan). After the measurements were completed, the oxygen administration method was changed according to the assigned order, and the measurements were repeated in the same manner.

### Statistical analyses

The results were analyzed using Friedman repeated measures analysis of variance by ranks with a post hoc Bonferroni test. All statistical analyses were performed with R (The R Foundation for Statistical Computing, Vienna, Austria). Differences were considered significant at a *P* value of < 0.05. Analysts were blinded to the intervention method.

## Results

The intervention was provided to 37 patients, after excluding five patients for the following reasons: surgery was postponed in one patient, one patient did not require ICU admission after surgery, and no intervention was provided after admission in three patients due to a lack of human resources (Fig. 2). Patient background characteristics and the surgical procedures performed are shown in the online Supporting Information Table S1. Briefly, mean values of age, height, weight, and body mass index were 65.6 years, 164.0 cm, 63.7 kg, and 23.5 kg.m^−2^, respectively.

**Fig. 3:**
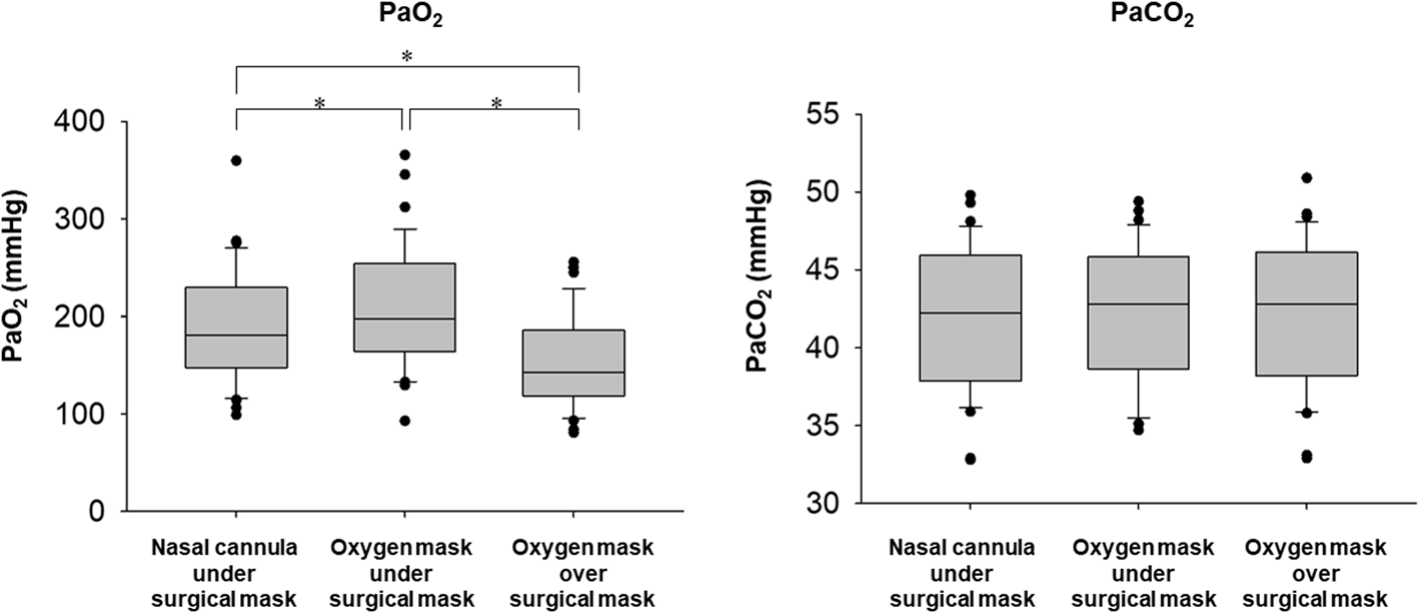
Comparison of partial pressure of arterial oxygen (PaO_2_) and partial pressure of arterial carbon dioxide (PaCO_2_) between the three oxygen administration methods. The ends of the box define the 25th and 75th percentiles, with the horizontal line in the middle showing the median, and error bars defining the 10th and 90th percentiles. The dots indicate outliers. *Friedman repeated measures analysis of variance by ranks with the post hoc Bonferroni test, *P* < 0.001

The measurement results of PaO_2_ and PaCO_2_ for each oxygen administration method are shown in Fig. 3. There was a significant difference in PaO_2_ between all three methods (Fig. 3A). Oxygenation was better when oxygen was administered via an oxygen mask worn under rather than over the surgical mask (median values 197.7 mmHg vs. 143.0 mmHg, *P* < 0.001), when oxygen was administered under the surgical mask with an oxygen mask rather than a nasal cannula (197.7 mmHg vs. 180.6 mmHg, *P* < 0.001), and when it was administered with a nasal cannula under the surgical mask rather than an oxygen mask over the surgical mask (180.6 mmHg vs. 143.0 mmHg, *P* < 0.001). No worsening of oxygenation meeting study discontinuation criteria was observed in any case and with any method. No differences in PaCO_2_ were found between each method (Fig. 3B). The median values of PaCO_2_ with oxygen administration via a nasal cannula under the surgical mask, with an oxygen mask under the surgical mask, and via an oxygen mask over the surgical mask were 42.2 mmHg, 42.8 mmHg, and 42.8 mmHg, respectively.

## Discussion

In the present study, we investigated the optimal method of oxygen administration with the simultaneous use of a surgical mask in postoperative patients after general anesthesia. When oxygen was administered at 4 L.min^−1^, PaO_2_ was higher with an oxygen mask used under the surgical mask, a nasal cannula under the surgical mask, and an oxygen mask over the surgical mask, in descending order. This study is the first randomized controlled trial examining the optimal method of administering oxygen during the simultaneous use of a surgical mask in patients after general anesthesia.

Several studies have examined the effects of surgical masks in combination with oxygen masks [7, 8, 11–13]. However, since these studies investigated the effects of surgical masks on inspired oxygen concentration, oxygenation was indirectly assessed. In the present study, PaO_2_ and PaCO_2_ were used as indices of oxygenation and ventilation to improve the accuracy of comparisons.

The results of this study suggested that oxygen administration with an oxygen mask over a surgical mask to prevent infection might result in worsening oxygenation. When oxygen is administered with an oxygen mask applied on top of a surgical mask, the inhaled oxygen concentration seems to drop, with a worsening of oxygenation. Thus, wearing an oxygen mask under the surgical mask might be more beneficial than wearing it over the surgical mask. This is consistent with our previous research on healthy subjects [10]. In the present study, PaCO_2_, an index of ventilation, was not affected by the oxygen administration method.

The present study and our previous study showed different results in terms of oxygenation when oxygen was administered using oxygen masks and nasal cannulas. Our previous study suggested that oxygen administration through a nasal cannula under a surgical mask is advantageous for oxygenation in healthy subjects. Possible causes for this discrepancy include the following: (1) differences in the balance between the mouth and nasal breathing by different study subjects (healthy volunteers vs. post-general anesthesia patients), (2) differences in the degree to which the mask was appropriately fitted to the face since we used a different oxygen mask in the present study as compared to our previous study (Figure S1). In this context, we conducted additional supplementary research to investigate the effects of the two oxygen masks on oxygenation. Figure S1 shows images of both types of oxygen masks, and the research methods are shown in the Supplemental methods.

Briefly, 24 healthy volunteers participated in our supplementary study, with no dropouts. The characteristics of the volunteers are shown in Table S2, and the flow of the study is shown in Figure S2. The study revealed differences in ORi between the two oxygen masks (Figure S3). When oxygen was administered under the surgical mask, the oxygen mask used in the present study showed greater ORi than the oxygen mask used in our previous study (median values 0.42 vs. 0.38, *P* = 0.003). Similarly, when oxygen was administered with the oxygen mask placed over the surgical mask, the mask used in the present study provided greater ORi than the mask used in our previous study (0.39 vs. 0.32, *P* = 0.004). Regarding the positional relationship between the oxygen mask and the surgical mask, ORi was greater when the oxygen mask was placed under the surgical mask than when it was placed over the surgical mask, regardless of the type of oxygen mask. This result is consistent with our previous report [10] and the present study on urological patients. The reversal of superiority in oxygenation between oxygen masks and nasal cannulas in patients and volunteers between our present and previous study is likely due to differences in the type of oxygen masks used in each study. However, since the additional study was conducted on healthy volunteers, the effect of different surgical masks on oxygenation might differ in patients. Therefore, we cannot rule out the possibility that other factors besides the difference in the type of oxygen masks might be responsible for the different results between patients and healthy volunteers regarding the superiority of oxygen masks and nasal cannulas for oxygenation. In any case, no patient in the present study was hypoxemic (median 180.6 mmHg, lower limit 99 mmHg) during oxygenation via a nasal cannula. In view of the results of the patient questionnaire, oxygen administration through a nasal cannula should be considered an alternative to oxygen administration using an oxygen mask under a surgical mask.

The present study has several possible limitations. First, the duration of each oxygen administration method might have been too short. Although the duration was sufficient to stabilize PaO_2_ and PaCO_2_, each method should have been used for a longer duration to adequately assess patient comfort. It is possible that the patient questionnaire results might change when the duration of oxygenation is extended to hours or days. Second, since the subject population was limited to urologic patients, generalizing the results might be difficult. The results might be different in patients who undergo thoracic surgery since the pain resulting from urologic wounds has minimal impact on respiratory movements. Third, this study only assessed differences in oxygen administration methods at an oxygen flow rate of 4 L.min^−1^. We chose a single oxygen flow rate to simplify the experimental protocol. Notably, although different flow rates might change the results, a similar study showed no significant difference when the oxygen flow rate was varied from 5 and 7 to 10 L.min^−1^ [8]. Fourth, we did not set up a group without a surgical mask in this study. Thus, we cannot comment on the possibility of benefits of oxygenation when a surgical mask is worn over an oxygen mask or nasal cannula compared to when it is not worn. Fifth, we did not examine how each method of oxygen administration prevented viral infections. Further research is needed to clarify this issue.

In conclusion, the current standard method of administering oxygen to postoperative patients using an oxygen mask over a surgical mask results in poor oxygenation. An alternative method of oxygen administration under the surgical mask should be considered in cases in which the patient’s oxygenation is poor with oxygen administration via an oxygen mask applied over the surgical mask.

## Supplementary Information

Supplementary Material 1: Table S1. Baseline characteristics of the patients. Table S2. Baseline characteristics of the healthy volunteers in the supplemental study. Table S3. Method of postoperative analgesia and analgesic agents used. Figure S1. Pictures of the oxygen masks used in the study. Figure S2. Subject recruitment, randomization and analysis in the supplemental study. Figure S3. Comparison of oxygen reserve index between the four oxygen administration methods. Supplemental methods: Research methods.

## Data Availability

The datasets used and/or analyzed during the current study are available from the corresponding author on reasonable request.
