# Supplementary material for: Investigation of the optimal method of oxygen administration with simultaneous use of a surgical mask in postoperative patients: a randomized cross-over study
Source: JA Clin Rep. 2024 Sep 28;10:60. doi: 10.1186/s40981-024-00741-0 (PMC11438753; doi:10.1186/s40981-024-00741-0)
Supplement: Supplementary file 1 — Supplementary Material 1: Table S1. Baseline characteristics of the patients. Table S2. Baseline characteristics of the healthy volunteers in the supplemental study. Table S3. Method of postoperative analgesia and analgesic agents used. Figure S1. Pictures of the oxygen masks used in the study. Figure S2. Subject recruitment, randomization and analysis in the supplemental study. Figure S3. Comparison of oxygen reserve index between the four oxygen administration methods. Supplemental methods: Research methods. [file 40981_2024_741_MOESM1_ESM.docx]

Supporting Information

**Table S1**

Baseline characteristics of the patients

|  | Total (n = 37) | Male (n =29) | Female (n =8) |
| --- | --- | --- | --- |
| Age (yr) | 65.6 ± 11.5 | 66.0 ± 10.0 | 64.4 ± 16.6 |
| Height (cm) | 164.0 ± 7.9 | 166.8 ± 6.2 | 154.1 ± 4.6 |
| Weight (kg) | 63.7 ± 12.6 | 66.7 ± 11.4 | 53.0 ± 11.1 |
| Body mass index (kg.m^-2^) | 23.5 ± 3.7 | 23.9 ± 3.5 | 22.3 ± 4.3 |

The surgical procedures performed in the patients were as follows:

- robot-assisted laparoscopic partial nephrectomy: 14 cases
- robot-assisted laparoscopic radical prostatectomy: eight cases
- laparoscopic nephrectomy: five cases
- total cystectomy and creation of an ileal conduit: four cases
- retroperitoneoscopic adrenalectomy: three cases
- open nephrectomy: two cases
- retroperitoneoscopic resection of a retroperitoneal tumor: one case

**Table S2**

Baseline characteristics of the healthy volunteers in the supplemental study

|  | Total (n = 24) | Male (n = 12) | Female (n = 12) |
| --- | --- | --- | --- |
| Age (yr) | 33.8 ± 8.4 | 30.0 ± 3.8 | 37.5 ± 10.1 |
| Height (cm) | 165.5 ± 7.6 | 171.9 ± 4.7 | 160.1 ± 5.9 |
| Weight (kg) | 60.0 ± 10.8 | 67.3 ± 8.8 | 52.7 ± 7.3 |
| Body mass index (kg.m^-2^) | 21.8 ± 2.9 | 23.0 ± 2.5 | 20.6 ± 2.9 |

**Table S3**

Method of postoperative analgesia and analgesic agents used

| Patient No. | Method of analgesia | Analgesic agents filled in the infuser | Dosing rate (ml/h) | Adjunctive analgesic agents |
| --- | --- | --- | --- | --- |
| 1 | IV-PCA | Fentanyl 1.5 mg, saline 28 ml, droperidol 5 mg | 1 | Acetaminophen 1000 mg ×2, flurbiprofen axetil 50 mg |
| 2 |  | Fentanyl 1.5 mg, saline 28 ml, droperidol 5 mg | 1 |  |
| 3 |  | Fentanyl 1.5 mg, saline 28 ml, droperidol 5 mg | 1 |  |
| 4 |  | Fentanyl 1.5 mg, saline 28 ml, droperidol 5 mg | 1 |  |
| 5 |  | Fentanyl 1.5 mg, saline 28 ml, droperidol 5 mg | 1 |  |
| 6 |  | Fentanyl 1.5 mg, saline 28 ml, droperidol 5 mg | 1 |  |
| 7 |  | Fentanyl 1.5 mg, saline 30 ml | 1 |  |
| 8 |  | Fentanyl 1.5 mg, saline 30 ml | 1 | Acetaminophen 1000 mg |
| 9 |  | Fentanyl 1.5 mg, saline 28 ml, droperidol 5 mg | 1 |  |
| 10 |  | Fentanyl 1.5 mg, saline 30 ml | 1 | Continuous IV administration of fentanyl 25 μg/h |
| 11 |  | Fentanyl 1.5 mg, saline 28 ml, droperidol 5 mg | 1 |  |
| 12 |  | Fentanyl 1.5 mg, saline 30 ml | 1 |  |
| 13 |  | Fentanyl 1.5 mg, saline 30 ml | 1 |  |
| 14 |  | Fentanyl 1.5 mg, saline 28 ml, droperidol 5 mg | 1 |  |
| 15 |  | Fentanyl 1.5 mg, saline 28 ml, droperidol 5 mg | 1 |  |
| 16 |  | Fentanyl 1.5 mg, saline 30 ml | 1 |  |
| 17 |  | Fentanyl 1.5 mg, saline 30 ml | 1 | Continuous IV administration of fentanyl 25-50 μg/h, acetaminophen 1000 mg ×2 |
| 18 |  | Fentanyl 1.5 mg, saline 30 ml | 1 |  |
| 19 |  | Fentanyl 1.5 mg, saline 28 ml, droperidol 5 mg | 1 |  |
| 20 |  | Fentanyl 1.5 mg, saline 28 ml, droperidol 5 mg | 1 |  |
| 21 |  | Fentanyl 1.5 mg, saline 30 ml | 1 |  |
| 22 |  | Fentanyl 1.5 mg, saline 28 ml, droperidol 5 mg | 1 |  |
| 23 |  | Fentanyl 1.5 mg, saline 28 ml, droperidol 5 mg | 1 |  |
| 24 |  | Fentanyl 1.5 mg, saline 30 ml | 1 |  |
| 25 |  | Fentanyl 1.5 mg, saline 30 ml | 1 |  |
| 26 |  | Fentanyl 1.5 mg, saline 45 ml | 1 |  |
| 27 |  | Fentanyl 1.5 mg, saline 30 ml | 1 |  |
| 28 |  | Fentanyl 1.5 mg, saline 28 ml, droperidol 5 mg | 1 | Continuous IV administration of fentanyl 50 μg/h, acetaminophen 1000 mg, flurbiprofen axetil 50 mg |
| 29 |  | Fentanyl 1.5 mg, saline 30 ml | 1 |  |
| 30 |  | Fentanyl 1.5 mg, saline 29 ml, droperidol 2.5 mg | 1 |  |
| 31 |  | Fentanyl 1.5 mg, saline 30 ml | 1 |  |
| 32 | Epidural | 0.25% levobupivacaine 200 ml, fentanyl 0.5 mg, saline 90 ml | 6 |  |
| 33 |  | 0.25% levobupivacaine 200 ml, fentanyl 1 mg, saline 80 ml | 6 |  |
| 34 |  | 0.25% levobupivacaine 200 ml, fentanyl 1 mg, saline 80 ml | 4 |  |
| 35 |  | 0.25% levobupivacaine 200 ml, fentanyl 1 mg, saline 80 ml | 4 |  |
| 36 |  | 0.25% levobupivacaine 200 ml, fentanyl 1 mg, saline 79 ml, droperidol 2.5 mg | 6 |  |
| 37 |  | 0.25% levobupivacaine 200 ml, fentanyl 1 mg, saline 80 ml | 6 |  |

Of the 37 patients, 31 received intravenous patient controlled analgesia (IV-PCA) and six had catheters implanted in the epidural space. Adjunctive analgesic agents were administered within 24 hours of the patients’ admission to the ICU.

**Figure S1**

Pictures of the oxygen masks used in the study


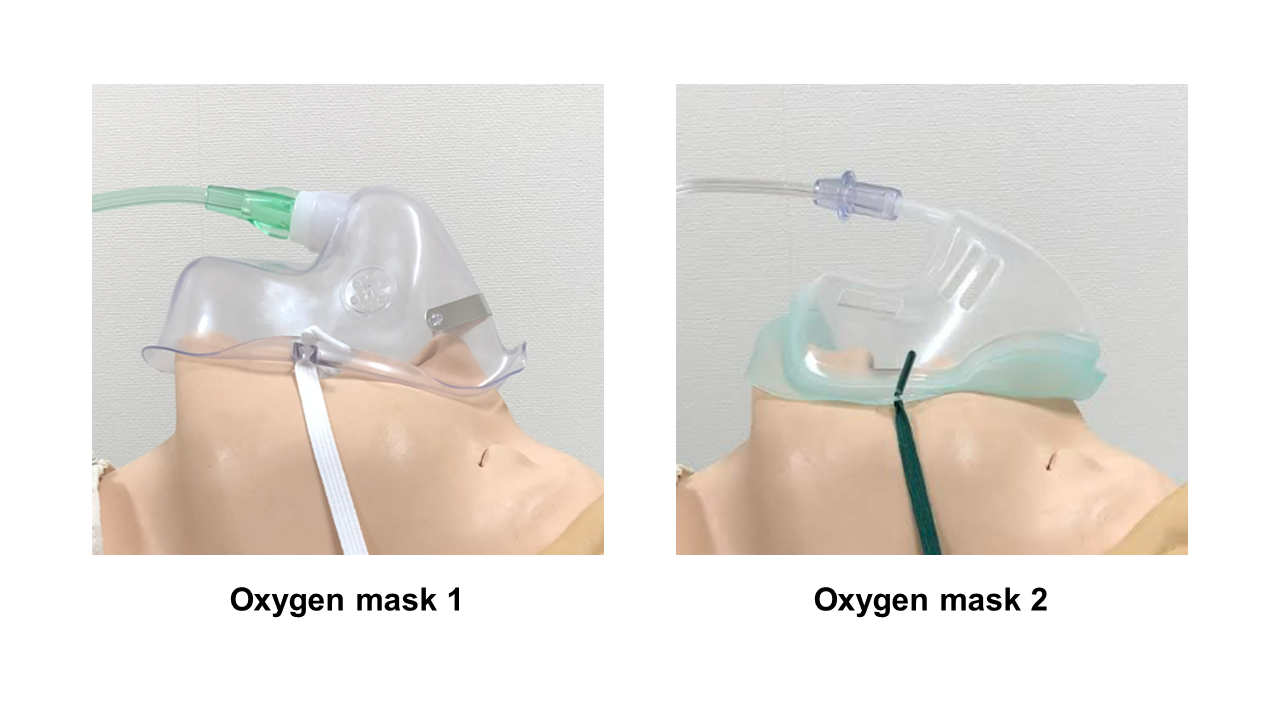
Oxygen mask 1 is the oxygen mask we used in our previous study on healthy subjects, and in the healthy volunteers in this additional study. Oxygen mask 2 is the oxygen mask used on postoperative patients in the present study.

**Figure S2**

Subject recruitment, randomization and analysis in the supplemental study


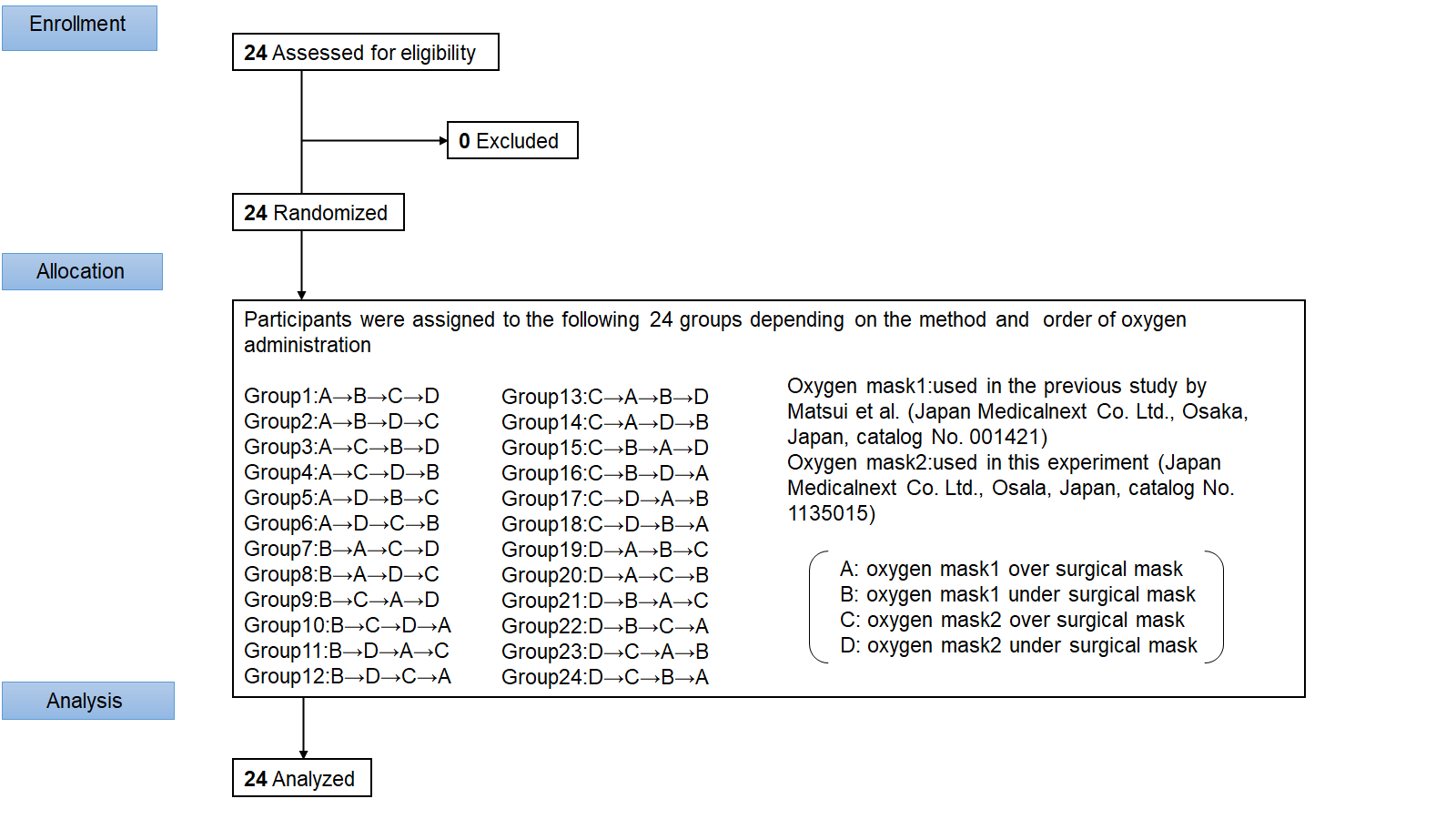


**Figure S3**

Comparison of oxygen reserve index between the four oxygen administration methods


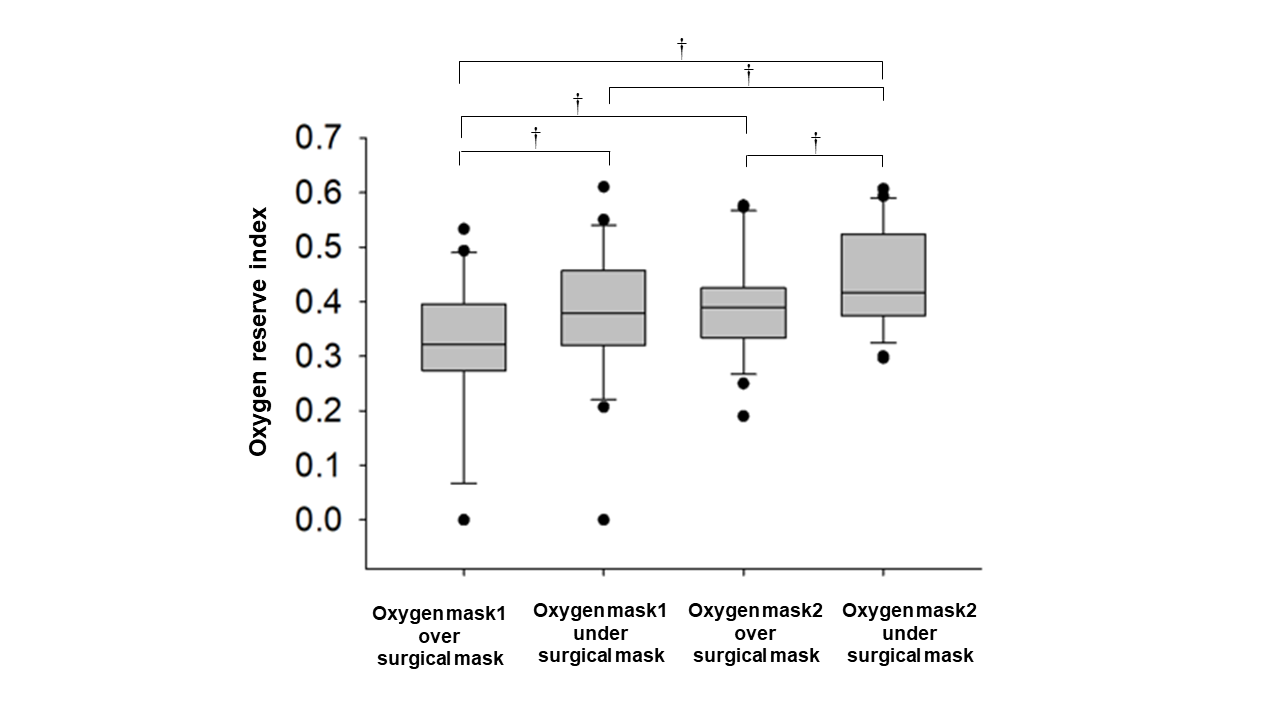


The ends of the box define the 25th and 75th percentiles, with the horizontal line in the middle showing the median, and the error bars defining 10th and 90th percentiles. The dots indicate outliers.

† Friedman repeated measures analysis of variance by ranks with the post hoc Bonferroni test, *P* < 0.01

**Supplemental Methods**

**Study design**

This randomized, single-blind, cross-over study was performed at the intensive care unit of Gunma University Hospital, from August 30, 2022, to September 14, 2022.

**Ethics approva**l

This study conformed to the standards of the Declaration of Helsinki and was approved by the ethics committee of Gunma University Hospital (Trial No. IRB2022-016). The study was registered with the University Hospital Medical Information Network Clinical Trials Registry (UMIN000048650) on August 15, 2022. The subjects participated on a voluntary basis, and written informed consent was obtained from each participant.

**Subjects**

Inclusion criteria were: (1) age between 20 and 60 years, and (2) legally competent to consent. Exclusion criteria were (1) history of respiratory illness, (2) smokers, (3) percutaneous arterial oxygen saturation of less than 92% before the measurement, and (4) exhaled carbon dioxide partial pressure of less than 28 mmHg or more than 45 mmHg. Based on our pilot study, the required sample size was 24 subjects with power analysis using *α* = 0.05 and *β* = 0.8. Twenty-four subjects were included in the study.

**Oxygen administration method**

Oxygen was administered at a flow rate of 4 L/min with two types of oxygen masks (Oxygen mask 1, Japan Medicalnext Co. Ltd., Osaka, Japan, catalog No. 001421, and Oxygen mask 2, Medicalnext Co. Ltd., catalog No. 1135015) (Supplemental Fig. 1). COMFORT+^®^ Level-1 (Medicom Japan Inc. Ltd., Kobe, Japan), which is the surgical mask we use in our daily practice and meets level 1 of the American Society for Testing and Materials (ASTM), was used as the surgical mask. Since two oxygen masks were used to administer oxygen either above or below the surgical mask, all subjects received oxygen in four different ways. The order of the four different oxygen administration methods was randomly selected from 24 possible order combinations. Assignments were made using the envelope method. Each volunteer received oxygen by each of the four methods in the assigned order.

**Measurement method and endpoints**

Oxygenation was evaluated using the oxygen reserve index (ORi) in a stable state under oxygen administration with each method.

The measurement method for ORi was the same as in our previous study [1, 2]: each oxygen mask was worn above or below the surgical mask, and the recorder was blind to the oxygen administration method due to the use of a blindfold and was single-blinded; ORi was measured noninvasively with a transcutaneous optical sensor using a Radical-7® pulse oximeter (Mashimo Corp., Irvine, CA, USA). The same person recorded the results in all cases. ORi and transcutaneous arterial blood oxygen saturation were measured at one and a half minutes or more after the start of oxygen administration, and at 10 seconds and 20 seconds thereafter, when oxygen levels were judged to have stabilized, and ORi was recorded as the average of the above three values.

**Statistical analyses**

The results were analyzed using Friedman repeated measures analysis of variance by ranks with a post hoc Bonferroni test. All statistical analyses were performed with R (The R Foundation for Statistical Computing, Vienna, Austria). Differences were considered significant at a *P* value of < 0.05. Analysts were blinded to the intervention method.

**References**

1. Matsui Y, Takazawa T, Takemae A, Saito S. Does a surgical mask improve oxygenation in COVID-19 patients? JA Clin Rep. 2021;7:34.
2. Matsui Y, Takazawa T, Takemae A, Murooka Y, Kanamoto M, Saito S. Investigation of the optimal method of oxygen administration with simultaneous use of a surgical mask: a randomized control study. J Anesth. 2022;36:26-31.
